# Vertical saccades and antisaccades: complementary markers for motor and cognitive impairment in Parkinson’s disease

**DOI:** 10.1038/s41531-019-0083-7

**Published:** 2019-06-24

**Authors:** Josefine Waldthaler, Panagiota Tsitsi, Per Svenningsson

**Affiliations:** 1 Section of Neurology, Department of Clinical Neuroscience, Center for Molecular Medicine, Karolinska Institute, Stockholm, Sweden; 2 University Hospital Marburg, Department for Neurology, Marburg, Germany

**Keywords:** Parkinson's disease, Diagnostic markers

## Abstract

Previous studies provide partly contradictory results about the characteristics of saccades in PD and the possible effects of levodopa, which may be attributed to different study design regarding disease stages, medication state or cognitive functioning. We studied horizontal and vertical visually guided saccades (VGS) and antisaccades (AS) in 40 patients with PD with and without postural instability in On and Off medication state as well as in 20 healthy controls (HC). Motor and cognitive performance were assessed using UPDRS, Montreal Cognitive Assessment (MoCA) and Frontal Assessment Battery (FAB). The PD group showed decreased VGS amplitudes and increased vertical VGS and AS latencies. Only relatively few studies had assessed vertical saccades in PD so far. However, our results indicate that vertical saccadic amplitude may be a supportive marker in diagnosing PD since upwards gain demonstrated an AUC of 0.85 for the discrimination of PD and HC. Only more advanced patients in Hoehn & Yahr stage 3 executed higher numbers of AS errors than HC. Since the AS error rate correlated with FAB and MoCA scores, AS performance seems to reflect cognitive ability in PD. Furthermore, the correlation of AS latency with the UPDRS axial subscore promotes the recently highlighted connection between postural control and executive function in PD. Levodopa did not alter saccade amplitudes and had opposing effects on the initiation of VGS and AS: Levodopa intake prolonged VGS latency, but decreased AS latency. Possible mechanisms by which levodopa may be capable of partially reversing the impaired balance between voluntary and reflexive cortical saccade initiation of PD are discussed.

## Introduction

Visual disturbances are common in Parkinson’s disease (PD)^1^ which is partly attributed to impaired oculomotor control. Saccades are the most studied eye movements since different patterns of saccade impairment reflect pathology in corresponding brain regions which, in turn, allows to distinguish between neurological conditions with similar symptoms but different pathophysiological substrate.^2^ For instance, analysis of saccades increases the accuracy in the differential diagnosis between PD and atypical Parkinsonian syndromes.^3^

Although the only clinical finding regarding eye movements in earlier stages of the disease may be a mild-upwards gaze palsy, surprisingly few studies assessed both horizontal and vertical saccades in the same session.^4^ Furthermore, previous studies trials provided contradictory data regarding several aspects of saccades in PD which may be attributed to different methodology and composition of study cohorts, such as various disease stages, ON vs. OFF medication state or exclusion of cognitively impaired patients.^5^ In PD patients, visually guided saccades (VGS) and voluntary saccades are hypometric^6^ which results in the need for multiple correction saccades to reach a visual target.^7^ This fragmentation of gaze shift is remarkable and has been proposed as a biomarker.^8^ Both, saccade amplitude and latency tend to worsen during the course of the disease^9^ whereby VGS latency is particularly prolonged in PD patients with mild cognitive impairment or dementia.^10^ PD patients make more directional antisaccade (AS) errors than healthy controls (HC).^11^ The performance in the AS task is related to functional and imaging markers of executive function in healthy elderly as well as patients with mild cognitive impairment.^12,13^ Recently, the same association was demonstrated in early, drug-naive PD patients.^14^ Ito et al. showed that the AS error rate correlates with Frontal Assessment Battery (FAB) score, but not to motor performance in PD.^15^

Previous studies found that levodopa administration decreases the latency of voluntary saccades, such as AS.^16^ However, the findings concerning improvement of saccadic amplitudes, VGS latency and AS error rate after levodopa intake are inconsistent.^14,16,17^

A critical region in the regulation of saccades is the superior colliculus (SC).^9^ Interestingly, recent anatomical and tracing data have provided evidence of a direct dopaminergic innervation of the SC from zona incerta.^18^ It remains to be determined whether this dopaminergic pathway is altered in PD, but we reasoned that it would be important to study patients OFF and ON levodopa to study the role of dopaminergic neurotransmission on saccades.

The objective of our study was to perform an analysis of horizontal and vertical saccades of 40 patients with PD, considering several aspects of the disease. To address the diverse disease severity, we decided to divide the PD group by means of postural control since recent studies pointed towards a relation between postural control and saccade performance in PD.^19^ Postural instability emerges with the transition from Hoehn and Yahr (H&Y) stage 2 to 3. Thus, 20 patients in H&Y2 and 20 patients in H&Y3 were included. The study may be divided in three parts: We aimed (1) to compare healthy controls and PD patients in off medication state with respect to disease stage, (2) to clarify the effects of levodopa intake on saccade performance, and (3) to evaluate potential correlations of these oculomotor parameters with motor and cognitive performance in PD.

## Results

### Saccades and levodopa

**Fig. 1:**
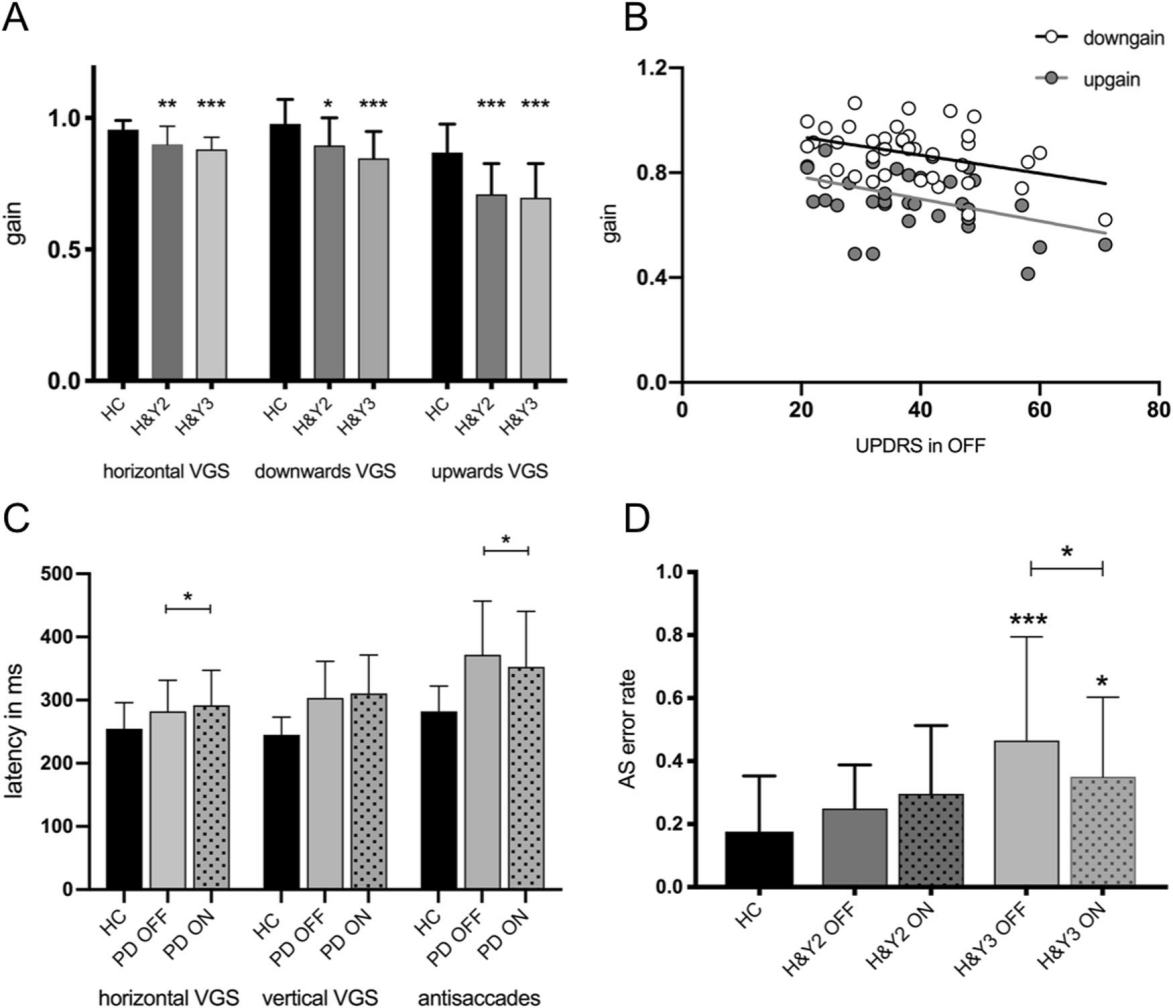
Levodopa effect on saccades. **a** VGS gain of healthy controls (HC), PD patients in H&Y2 and H&Y3 in OFF medication state. * indicate a significant group difference compared to healthy controls in the ANOVA; **b** linear regressions of vertical gain and UPDRS (upwards: *r* = −0.43; *p* = 0.01; downwards: −0.38; *p* = 0.02); **c** saccadic latency of HC and PD patients in OFF and ON medication state. The brackets show the results of the paired *t* tests comparing OFF and ON state. **d** AS error rates of HC and PD patients in H&Y2 and H&Y3 in OFF and ON medication state. The AS error rate was increased only in H&Y3 patients (ANOVA, compared to HC; * shown without brackets) and improved after levodopa in these patients (paired *t* test, shown with brackets). (*p ≤ 0.05, **p ≤ 0.01, ***p ≤ 0.005; error bars show standard deviation)

The analysis of variance (ANOVA) revealed an overall significant effect of group (HC, Hoehn and Yahr (H&Y)2, and H&Y3) for all VGS paradigms. Post-hoc multiple comparisons showed that the gain of upwards, downwards, and horizontal saccades was reduced in H&Y2 and in H&Y3 compared to HC (Table 1 for detailed results). Although the gain tended to be smaller in H&Y3 than in H&Y2 in all paradigms, no differences between the two PD groups reached significance (see Fig. 1a).

**Table 1:** Results of ANOVA comparing HC and PD patients in H&Y stage 2 and 3 (in off medication state)

	HC mean (SD)	H&Y2 mean (SD)	H&Y3 mean (SD)	*p* (ANOVA)
*Step horizontal*				
Latency (ms)	254.7 (40.3)	284.7 (58.6)	279.5 (34.1)	0.1
Gain	0.96 (0.03)	0.90 (0.07)**	0.88 (0.04)***	<0.0001
Step vertical				
Latency (ms)	245.3 (27.3)	303.8 (68.0)***	303.5 (42.8)***	0.0002
Downwards gain	0.98 (0.09)	0.89 (0.10)*	0.85 (0.10)***	0.0005
Upwards gain	0.87 (0.11)	0.71 (0.11)***	0.69 (0.13)***	<0.0001
*Antisaccades*				
Latency (ms)	282 (39.2)	374.5 (107.2)***	387.3 (58.1)***	0.0002
Express saccade rate	0.09 (0.12)	0.07 (0.11)	0.08 (0.15)	
AS error rate	0.18 (0.17)	0.25 (0.14)^a^	0.47 (0.32)***^a^	0.0003

^a^indicates a significant between-group difference between H&Y2 and H&Y3

Between-group differences compared to HC are shown as * using Dunn’s post hoc test with Bonferroni correction with a significance level of *p < 0.05, **p < 0.01, ***p < 0.005

The upwards and downwards gain were correlated with the total UPDRS III score (upwards: *r* = −0.43; *p* = 0.01; downwards: −0.38; *p* = 0.02; Fig. 1b), however, the correlations did not remain significant after correction for multiple testing (Table 2). We did not find any correlations of VGS gain with age, disease duration, or cognitive scores.

**Table 2:** Multiple linear correlations of saccade parameters with age, disease duration, UPDRS, and cognitive scores

	Age		Disease duration		UPDRS OFF	
OFF	*r*	*p*(FDR)	*r*	*p*(FDR)	*r*	*p*(FDR)
Horizontal VGS						
Gain	−0.12	0.90	0.00	0.98	0.00	0.98
Latency	0.29	0.38	0.01	0.98	−0.15	0.67
Vertical VGS						
Downwards gain	0.15	0.67	−0.07		−0.38	0.12
Upwards gain	−0.21	0.57	−0.08	0.88	−0.43	0.12
Latency	−0.02	0.98	0.16	0.67	0.24	0.57
Horizontal AS						
Latency	0.29	0.38	−0.05	0.98	0.16	0.67
Error rate	0.40	0.12	−0.10	0.88	0.08	0.88

	Age		Disease duration		UPDRS ON		Axial UPDRS		MoCA		FAB	
ON	*r*	*p*(FDR)	*r*	*p*(FDR)	*r*	*p*(FDR)	*r*	*p*(FDR)	*r*	*p*(FDR)	*r*	*p*(FDR)
Horizontal VGS												
Gain	−0.13	0.65	−0.08	0.75	−0.12	0.61	−0.22	0.34	0.20	0.36	0.30	0.19
Latency	0.29	0.19	0.01	0.97	0.05	0.90	0.19	0.41	0.02	0.94	−0.04	0.90
Vertical VGS												
Downwards gain	0.21	0.36	−0.25	0.29	−0.29	0.19	−0.26	0.25	0.21	0.36	0.22	0.36
Upwards gain	−0.15	0.50	−0.12	0.61	−0.34	0.16	−0.33	0.16	0.09	0.71	0.40	0.41
Latency	0.29	0.19	0.17	0.44	0.04	0.90	0.31	0.19	−0.04	0.90	0.02	0.94
Horizontal AS												
Latency	0.30	0.19	0.24	0.31	0.40	0.10	0.44	0.043*	−0.18	0.41	−0.34	0.16
Error rate	0.44	0.053	0.14	0.54	0.37	0.15	0.34	0.16	−0.51	0.018	−0.72	0.0041**

The corrected *p* value *p*(FDR) was calculated using the false-discovery rate (FDR) method

*p < 0.05, **p < 0.01

Using ROC, upwards gain showed the greatest AUC of all saccade parameters for the discrimination of PD patients from HC individuals (0.85; 95% CI: 0.75–0.95, *p* < 0.0001). A cut-off gain of <0.755 resulted in an optimal sensitivity of 68% and specificity of 90%.

VGS gain was not improved by levodopa intake in any paradigm (Table 3).

**Table 3:** Within-subject comparison with paired *t* test in off and on medication state (*n* = 40)

	PD OFF mean (SD)	PD ON mean (SD)	*p* (paired *t* test)
Step horizontal			
Latency (ms)	282.2 (48.6)	291.9 (54.7)	0.03*
Gain	0.89 (0.06)	0.88 (0.08)	0.6
Step vertical			
Latency (ms)	303.7 (57.2)	310.6 (60.3)	0.2
Downwards gain	0.87 (0.10)	0.72 (0.13)	0.4
Upwards gain	0.70 (0.12)	0.69 (0.13)	0.8
Antisaccades			
Latency (ms)	372.0 (83.6)	352.8 (86.6)	0.04*
Express saccade rate	0.07 (0.11)	0.08 (0.15)	0.4
AS error rate	0.35 (0.27)	0.32 (0.23)	0.5

*p < 0.05

Latency of vertical VGS, but not horizontal VGS, was significantly increased in H&Y2 and H&Y3 compared to HC (Table 1). The VGS latencies did not show any significant differences between H&Y2 and H&Y3, nor correlations with age, cognitive, or motor score.

The latency of horizontal VGS increased significantly after levodopa intake (Fig. 1c).

AS latency was prolonged in H&Y2 and H&Y3 compared to HC. AS error rate of H&Y2 patients did not differ from HC, while H&Y3 patients showed an increased AS error rate compared to both, H&Y2 and HC.

Levodopa administration led to a significant reduction of AS latency, but not of AS error rate, when the whole-PD sample was included into the analysis. Here, we conducted a post hoc analysis by H&Y stage: H&Y2 patients showed a significant decrease of AS latency after levodopa intake (*p* = 0.02), while the AS latency remained unchanged in H&Y3 patients (*p* = 0.6). Instead, levodopa did improve AS error rate in H&Y3 patients (*p* = 0.03; Fig. 1d).

AS latency was significantly correlated with the axial UPDRS subscore. No further correlations of AS latency remained significant after correction for multiple testing (see Table 2 for detailed results). AS error rate in ON correlated negatively with Montreal Cognitive Assessment (MoCA) and FAB. AS error rate tended to a correlation with age, total UPDRS and axial UPDRS subscore (Table 2). The correlations of AS error rate with FAB remained significant in a linear regression model with age as a co-variable (*t* = −5.13; *R*^2^ = 0.49; *p* < 0.001).

## Discussion

In this study, we examined the saccade performance of 40 PD patients in the context of several clinical aspects of the disease including motor stage, postural stability, medication state, cognitive function. In the following sections, we want to discuss our results which, in summary, indicate that saccadic gain may primarily reflect motor burden in PD while the saccadic latency is additionally modified by higher cognitive control.

The gain of VGS of all gaze directions was reduced in PD patients with upwards hypometria being most prominent. Some early studies suggested that the gain of VGS may be within the normal range in PD.^11^ A reason for conflicting findings regarding VGS gain in PD in the literature may be caused by the inclusion of patients cohorts with differing disease severity. However, our findings support a growing body of evidence^20^ that saccadic hypometria is present in mild and moderate stage PD, beginning at least in H&Y stage 2.

As the technical properties of eye trackers have improved, the assessment of vertical saccades should no longer be neglected in studies of eye movements in PD for methodological reasons. A mean upwards gain of less than 0.75 reached a specificity of 90% in discriminating PD and HC. Although the sensitivity of 68% was rather low to be clinical useful if standing alone, applying machine-learning approaches may appreciably improve the diagnostic accuracy by incorporating more than one eye tracking parameter into a diagnostic algorithm in the future. Then, vertical saccadic gain may also be promising in the early diagnosis of PD. Studies investigating drug-naïve, newly diagnosed patients and persons at risk with symptoms of prodromal PD are needed to determine the usefulness of vertical gain as an early marker for motor burden in PD.

In PD, the reduced amplitude of saccades is most likely caused by dual excessive suppression of the superior colliculus by (1) a direct downstream inhibition via thalamus by the internal segment of globus pallidus and substantia nigra and (2) a deteriorated pre-oculomotor drive through the frontal—basal ganglia circuit, e.g., the indirect pathway.^20^ Recent data even suggests a direct dopaminergic innervation of the superior colliculus from zona incerta.^18^ It is still surprising that levodopa does not ameliorate the saccadic hypometria in PD, even if it had a clinically sufficient effect on motor performance, reflected by a appropriate decrease of UPDRS in our cohort. Thus, the discussed dopaminergic mechanisms may not be exhaustive explanations for the saccadic hypometria in PD.

Horizontal saccades have been disproportionally more often investigated in PD studies than vertical saccades which is partly due to technical challenges when measuring vertical eye movements. In addition, the network of horizontal saccade execution is well described which facilitates the interpretation of the results of the studies: the contralateral parietal and frontal eye fields (PEF, respectively FEF) are the cortical areas primarily involved in horizontal saccade generation.^21^ The circuits needed to execute vertical saccades seem to be more complex, as a functional magnetic resonance imaging (fMRI) study of vertical saccades in PD and HC showed that vertical reflexive saccades cause a higher level of activation in the right FEF, cerebellar posterior lobe, and superior temporal gyrus compared to horizontal saccades.^4^ In contrast to horizontal VGS, we found no change in the vertical VGS latency after levodopa intake, supporting the functional relevance of a higher impact of dopamine-independent pathways in the initiation of vertical saccades.

Levodopa increases the latency of horizontal VGS, while it decreases the latency of AS in PD. The opposing effects of levodopa on reflexive and voluntary saccadic latencies are a recurring, but not completely understood finding in eye movement studies of PD.^9,16,20^ This may seem like a contradiction at first glance. In the following section, we attempt to theoretically dissolve this paradox based on the different roles of parietal and frontal eye fields in reflexive and voluntary saccade generation. Highly reflexive VGS, like those performed in the setting of our study, are triggered mainly by PEF bypassing basal ganglia circuits.^22^ AS, however, are volitional and two mechanisms are mandatory for their execution: (1) the top-down inhibition of a reflexive saccade towards the target provided by the dorsolateral prefrontal cortex (DLPFC) and (2) triggering of an intentional saccade to the opposite direction provided by the FEF.^23^ In addition to a direct circuit between FEF and SC, FEF is part of a reciprocal loop with the basal ganglia.^24^ The enhanced inhibiting output of basal ganglia in PD suppresses frontal areas of oculomotor generation, while it may have less impact on PEF. In fMRI studies, PD patients showed remarkable hypoactivity in frontal and supplementary eye fields with normal activation of PEF during horizontal voluntary saccades.^21^

In OFF medication state, the suppression of frontal areas by basal ganglia may cause reduced evaluative processing before a reflexive saccade is triggered by PEF, resulting in a slightly shorter latency of VGS. After levodopa intake, the balance between the indirect and direct basal ganglia pathways shifts in favor to the latter. In turn, the inhibitory output of basal ganglia on FEF and DLPFC decreases in ON medication state. Subsequently, an increase of frontal-derived evaluative processes concerning target selection and decision making may lead to the slight increase of VGS latency before a saccade is triggered by PEF.

In contrast to VGS, the initiation of AS is seen as a primarily frontal-based task provided by FEF and DLPFC.^24^ Based on our model, levodopa administration leads to a reduced inhibition of prefrontal areas by the basal ganglia, thereby to a facilitation of the processing required for inhibition of a VGS which, in turn, results in the decreased AS latency. Thus, levodopa may be capable of reversing the impaired balance between the voluntary and reflexive system of saccade generation in PD. However, this hypothesis demands confirmation in functional imaging studies.

In line with several studies in individuals with normal cognition, amnestic MCI and PD,^12–14^ we found a correlation of the AS error rate with the performance in the FAB, a neuropsychological test assessing executive function and inhibition control. Although the frontal dysfunction, resulting in the specific cognitive profile of PD is a well-known non-motor feature, earlier studies showed diverging results regarding alterations of AS error rate and AS latency in PD.^14,16,25^ One reason for the contradictory findings may have been that disease stages and medications states differed between the studies. Both seem to be important factors contributing to AS performance, as indicated by our results: in H&Y2 patients, the AS error rate did not differ from HC, but the AS latency was prolonged in OFF medication state and improved after levodopa intake. In contrast, H&Y3 patients in the more advanced stage of the disease showed a higher AS error rate which was significantly corrected after levodopa intake.

AS latency showed a trend toward a correlation with the axial UPDRS subscore and AS error rate with total UPDRS which, however, did not remain significant after correction for multiple testing. One may argue that this correlation may be more likely a result of a deterioration of both factors with disease progression than of a direct relationship. However, a recent study of AS in PD patients with postural instability demonstrated similar results. Here, the AS latency correlated with the stand-walk-sit time and the duration of anticipatory postural adjustments before gait initiation.^26^ Given that postural instability is strongly associated with cognitive decline and executive dysfunction in particular,^26,27^ our results may suggest that AS performance could have the potential to help predicting subjects at risk of falls.

It is of notice, that patients in H&Y3 were not only more impaired in motor functions and axial symptoms. They were also slightly older and may have been more affected by cognitive decline, although their performance in MoCA and FAB did not differ from the patients in H&Y2. Since a comprehensive neuropsychological testing was not included in this study, we cannot exclude this bias completely.

Some additional limitations of our study need to be discussed. First, the sample size is smaller than in some previous studies.^9^ An extension of the study population to the early and late stages of PD would have been interesting. We refrained from the inclusion of patients in H&Y 4 and 5 as we gave preference to the assessment in OFF medication state which might not have been practicable for many of these patients. Some patients were taking dopamine agonits alongside levodopa. Although those medications were stopped for 12 hours prior to the OFF session, they may have had an influence on saccade performance due to their long half-life.

The differentiation between primary oculomotor deficits and impaired higher cognitive processing is challenging in studies of eye movements in PD, especially since the pathophysiological mechanisms are not fully understood yet. Imaging studies using fMRI, PET or MEG will contribute to a broader understanding of the underlying functional networks in the future. As our results demonstrate, a careful characterization of the cohort regarding cognition, medication state and disease stage is crucial in eye tracking studies of PD to avoid more conflicting data in the field.

## Methods

### Study population

The study population consisted of consecutive non-demented 40 PD patients, 20 in H&Y 2 and 20 in H&Y 3, and 20 age-matched HC. For demographic data and clinical scores, see Table 4.

**Table 4:** Clinical data for HC and PD patients (left), respectively patients in H&Y2 and H&Y3 (right)

	HC	PD	*p* (*t* test)	H&Y2	H&Y3	*p* (paired *t* test)
*n*	20	40		20	20	
Gender (male/female)	12/8	28/12		15/5	13/7	
Age in years	65.9 (7.6)	65.6 (9.1)	0.2	62.2 (9.0)	69.2 (7.7)	0.01*
MoCA	26.9 (1.9)	25.3 (3.7)	0.04*	26.0 (3.1)	24.6 (4.1)	0.3
FAB	16.7 (1.4)	15.5 (2.7)	0.045*	15.8 (2.8)	15.3 (2.5)	0.6
Disease duration in years		4.9 (3.4)		3.5 (2.5)	6.3 (3.6)	0.007**
Levodopa equivalent dose in mg per day		528 (235)		479 (231)	580 (227)	0.2
MDS-UPDRS III OFF		38.4 (11.6)		34.1 (9.8)	42.9 (11.6)	0.02*
MDS-UPDRS III ON		25.5 (10.8)		21.0 (9.6)	30.5 (9.8)	0.004***
Axial UPDRS subscore OFF		7.4 (3.0)		5.7 (2.2)	9.2 (2.8)	0.0003***
Axial UPDRS subscore ON		5.1 (2.8)		3.6 (1.9)	6.7 (2.7)	0.0005***

Data are presented as mean (standard deviation). *p < 0.05, **p < 0.01, ***p *<* 0.005

The study was approved by the local ethical committee (2016/348-31/4) of Karolinska University Stockholm and all participants gave written informed consent.

### Clinical assessment

PD patients were examined twice: after withdrawal of all dopaminergic medication for at least 12 h (practical OFF medication state) and 1 h after intake of their individual regular medication (ON medication state). Part 3 of the Movement Disorders Society-revised Unified Parkinson's Disease Rating Scale (MDS-UPDRS part III, hereafter UPDRS) was used for assessment of motor symptoms. In addition, we calculated an axial (posture, gait, arising from a chair, postural stability, speech, and nuchal rigidity) subscore, as proposed in ref. ^19^

The cognitive assessment using MoCA and FAB was performed in ON medication state.

### Eye tracking procedures

Eye movements were analyzed using EyeBrain T2 (medical device with CE label for clinical use Class IIa, ISO 9001, ISO 13485), a head-mounted binocular eye-tracker that tracks the pupil using near-infrared light and an acquisition speed at 300 Hz. Data were acquired for both eyes by presenting stimuli on a 22-in wide screen 60 cm away using MeyeParadigm^®^ 2.1. A chin rest minimized head movement during recording.

For each paradigm, a series of 12 stimuli was given after standardized verbal instructions. Each stimulus appeared from a central target position outward for a fixed period of 1000 ms. Paradigms, included VGS with fixed target amplitudes in a horizontal (20°) and vertical (12°) step task and horizontal AS (20°). Saccades were identified as eye movements with a minimum velocity of 100° VA/s and a minimum duration of 16 ms. Measured parameters were saccadic latency, mean and peak velocity, gain, AS error rate as well as rates of express and anticipated saccades. Express and anticipated saccades (<135 ms) as well as saccades with directional errors were excluded from analysis of latency. Regarding vertical saccades, we assessed upwards and downwards gain separately. Since we did not find any differences in average saccadic velocity between PD patients and controls, the results are not shown.

### Statistical analysis

Normal distribution of data was tested with Shapiro–Wilk test. All reported *p* values are two-tailed and a *p* value < 0.05 was considered significant. Statistical analysis was performed in Prism 8 (Graph Pad).

Part one of the analysis was to compare healthy controls and PD patients in off medication state with different disease severity. Differences in saccade parameters between HC and PD patients in H&Y2 and H&Y3 in OFF medication state were evaluated using ANOVA followed by Dunn’s post hoc test with Bonferroni correction.

The aim of the second part was to investigate effect of levodopa effect on saccade parameters. Here, we performed paired *t* tests to compare ON and OFF states within PD subjects.

Furthermore, Pearson’s correlations with false-discovery rate correction for multiple testing were used to examine potential associations between saccade parameters, age, UPDRS, MoCA and FAB. Since neuropsychological assessment was performed in ON medication state, correlations for cognitive scores were only calculated for this motor state.

### Reporting summary

Further information on experimental design is available in the Nature Research Reporting Summary linked to this article.

## Supplementary Information

Supplementary Information

## Data Availability

The datasets generated and analyzed during this study are available from the corresponding author on reasonable request.
